# Genomic Screening at a Single Health System

**DOI:** 10.1001/jamanetworkopen.2025.0917

**Published:** 2025-03-17

**Authors:** Juliann M. Savatt, Melissa A. Kelly, Amy C. Sturm, Cara Z. McCormick, Marc S. Williams, Michelle Pistner Nixon, David D. Rolston, Natasha T. Strande, Karen E. Wain, Huntington F. Willard, W. Andrew Faucett, David H. Ledbetter, Adam H. Buchanan, Christa L. Martin

**Affiliations:** 1 Geisinger, Danville, Pennsylvania; 2 23andMe, Sunnyvale, California; 3 Division of Laboratory Genetics and Genomics, Department of Laboratory Medicine and Pathology, Mayo Clinic, Rochester, Minnesota; 4 Genome National Group, Durham, North Carolina; 5 Office of Research Affairs, Departments of Pediatrics and Psychiatry, University of Florida College of Medicine–Jacksonville

## Abstract

**Importance:**

Completion of the Human Genome Project prompted predictions that genomics would transform medicine, including through genomic screening that identifies potentially medically actionable findings that could prevent disease, detect it earlier, or treat it better. However, genomic screening remains anchored in research and largely unavailable as part of routine care.

**Objective:**

To summarize 11 years of experience with genomic screening and explore the landscape of genomic screening efforts.

**Design, Setting, and Participants:**

This cohort study was based in Geisinger’s MyCode Community Health Initiative, a genomic screening program in a rural Pennsylvania health care system in which patient-participants exomes are analyzed.

**Main Outcomes and Measures:**

Genomic screen-positive rates were evaluated and stratified by condition type (cancer, cardiovascular, other) and US Centers for Disease Control and Prevention (CDC) Tier 1 designation. The proportion of participants previously unaware of their genomic result was assessed. Other large-scale population-based genomic screening efforts with genomic results disclosure were compiled from public resources.

**Results:**

A total of 354 957 patients participated in Geisinger’s genomic screening program (median [IQR] age, 54 [36-69] years; 194 037 [59.7%] assigned female sex at birth). As of June 2024, 175 500 participants had exome sequencing available for analysis, and 5934 participants (3.4%) had a pathogenic variant in 81 genes known to increase risk for disease. Between 2013 and July 2024, 5119 results were disclosed to 5052 eligible participants, with 2267 (44.2%) associated with risk for cardiovascular disease, 2031 (39.7%) with risk for cancer, and 821 (16.0%) with risk for other conditions. Most results (3040 [59.4%]) were in genes outside of those with a CDC Tier 1 designation. Nearly 90% of participants (4425 [87.6%]) were unaware of their genomic risk prior to disclosure. In a survey of large-scale biobanks with genomic and electronic health record (EHR) data, only 25.0% (6 of 24) disclosed potentially actionable genomic results.

**Conclusions and Relevance:**

In this large, genomics-informed cohort study from a single health system, 1 in 30 participants had a potentially actionable genomic finding. However, nearly 90% were unaware of their risk prior to screening, demonstrating the utility of genomic screening in identifying at-risk individuals. Most large-scale biobanks with genomic and EHR data did not return genomic results with potential medical relevance, missing opportunities to significantly improve genomic risk ascertainment for these individuals and to perform longitudinal studies of clinical and implementation outcomes in diverse settings.

## Key Points

**Question:**

Does genomic screening increase identification of disease risk, and what is the current landscape of genomic screening programs?

**Findings:**

In this cohort study, genomic screening of 175 500 patient-participants from a health care system–based biobank revealed 3.4% with a potentially medically actionable result; results were disclosed to 5052 participants, nearly 90% of whom were unaware of their genomic risk. Of 24 biobanks meeting size and genomic data availability inclusion criteria, only 6 (25%) disclosed potentially actionable genomic results.

**Meaning:**

In this study, genomic screening identified potentially actionable results in 1 in 30 individuals, demonstrating its utility in identifying disease risk; however, its application remains limited, representing missed opportunities to ascertain at-risk individuals.

## Teaser

This cohort study reports on a large-scale and long-term genomic screening program, evaluating the percentage of actionable results discovered and disclosed to participants.

## Introduction

Completion of the Human Genome Project was lauded with predictions that genomics would transform medicine by using individuals’ DNA sequences to identify their disease risk. Twenty years later, our understanding of the relationship between genomics and health has dramatically increased, and sequencing costs have plummeted. In addition, there is growing evidence that genomic screening for potentially medically actionable findings, defined as those that can prompt medical care to prevent, delay, or reduce symptoms (commonly referred to as *actionable findings* in the literature^1^), can lead to impactful changes in medical care.^2^

For example, a male in his forties only learned of his increased genomic risk for medullary thyroid cancer, caused by a pathogenic variant in the *RET* gene, through participation in Geisinger’s genomic screening program.^3^ He did not have any personal or family history warranting phenotype-driven genetic testing. His genomic result prompted imaging that revealed a thyroid nodule. Although a biopsy was benign, the patient elected for thyroidectomy because of his genomic risk, revealing a medullary thyroid microcarcinoma. Genomic screening empowered early cancer detection and enabled identification of at-risk relatives.

This participant’s story and additional data from this genomic screening program and other similar initiatives highlight the power of genomic screening to identify at-risk patients, enable relevant diagnoses, and prompt changes to care.^4,5,6,7,8,9,10,11,12^ Despite these findings, genomic screening remains largely anchored in research, with few studies prioritizing disclosure of potentially medically actionable results to participants. Furthermore, the translation of genomic screening into routine clinical care has been slow, missing opportunities to identify at-risk patients for tailored, preventive care. Currently, identification of individuals with genomic risk remains largely dependent on clinical testing that relies on a personal and/or family history of disease and access to specialty care.

Here, we report our 11-year experience from a health care system–based genomic screening program that includes patients who agreed to participate and that has now sequenced more than 175 000 participants and disclosed more than 5000 genomic results. We also explore the landscape of other genomic screening efforts to highlight opportunities to integrate disclosure of potentially actionable genomic results.

## Methods

### Geisinger’s Genomic Screening Program

Geisinger is a nonprofit integrated health care system in rural central and northeast Pennsylvania serving approximately 1 million active patients. Launched in 2007, MyCode was envisioned as a biobank linking patient-participant biological samples and electronic health records (EHR) to enable translational research.^3^ Participants are invited to consent to the program regardless of phenotype or family history, building a health system–based cohort. In 2013, anticipating the addition of genomic information to the program, consent was updated to include disclosure of potentially medically actionable results. Through a collaboration with Regeneron Genetics Center that began in 2014, research exome sequencing has been completed for a subset of participants.^13^ The first genomic results were disclosed to participants in 2015.^3^

The genomic screening program, the genomic results disclosure process, and this study were approved by the Geisinger institutional review board. Informed consent for the genomic screening program was obtained from all participants; EHR data were collected under an exempt protocol. This report follows the Strengthening the Reporting of Observational Studies in Epidemiology (STROBE) reporting guideline for cohort studies.

We have developed and implemented a process for genomic screening and disclosure of potentially actionable genomic results that leverages participants’ research exome data.^14,15^ Genes designated for disclosure undergo regular assessment.^15^ The gene list for disclosure mirrors the American College of Medical Genetics and Genomics (ACMG) Secondary Finding (SF) version 3.2 recommendation^16^ (eTable 1 in Supplement 1). Variants are clinically confirmed in a College of American Pathologists/Clinical Laboratory Improvement Amendments clinical laboratory, and only pathogenic/likely pathogenic (P/LP) variants are disclosed; variants of uncertain significance are not. Results are uploaded into each participant’s EHR, and the primary care clinician and participants are notified of the result and given information about the condition and management resources. The patient-participant is also offered genetic counseling.^14,17^ Costs of clinical confirmation, disclosure, and initial genetic counseling are covered by the program.

The rate of screen-positive results among participants with exome sequences available for analysis (n = 175 500) as of July 2024 were assessed. Participants’ age; sex assigned at birth; and self-reported, EHR-documented race and ethnicity were extracted from the EHR in September 2024. Due to historical offering of the genomic screening program at collaborating health systems, demographic data are missing on a subset (n = 30 012) of participants; actionable genomic findings were disclosed to eligible participants from this subset with exome sequences available for analysis (8322 sequences) and with P/LP variants. Race and ethnicity data were collected and analyzed to report the characteristics of the cohorts and inform the generalizability of results. Characteristics of participants with results disclosed were compared with those of participants with sequencing available but without results and with those of the remaining cohort without sequencing data. For participants with disclosed results, the proportion of participants previously unaware of their genomic risk was assessed. Data on participants’ results and prior knowledge of their results were compiled through EHR queries and participant report during disclosure. Results were categorized based on condition (cancer, cardiovascular, other) and US Centers for Disease Control and Prevention (CDC) Tier 1 designation^18^ (eTable 1 in Supplement 1). Rates of participants previously aware of their genomic result were compared across condition type.

### Genomic Screening Programs

To explore the current landscape of large-scale genomic screening efforts and their disclosure of potentially medically actionable findings, we compiled information in April 2024 from noncommercial programs with more than 100 000 participants that had EHR and exome or genome sequence data available for at least a subset of participants. We denoted which programs were actively returning genomic results. Reasons for not disclosing genomic screening results were not assessed. Data were collected from review of the International Health Cohorts Consortium,^19^ the literature, and program websites.

### Statistical Analysis

Statistical hypothesis tests (α = .05) were used to compare groups. Two-sample *t* tests were used to compare means between groups, and χ^2^ tests or 2-proportion *z* tests were used to compare proportions between groups. Participants with missing demographic information were excluded from hypothesis testing involving relevant variables. Result type by disease area and CDC Tier 1 status were compared among participants with demographic information and those without (eTables 2 and 3 in Supplement 1). While there were no significant differences in result type between missingness of sex and these variables, there were significant differences between missingness of race and ethnicity and CDC Tier 1 status. All analyses were conducted in R version 4.4.1 (R Project for Statistical Computing).

## Results

### Geisinger’s Genomic Screening Program

**Figure 1.**
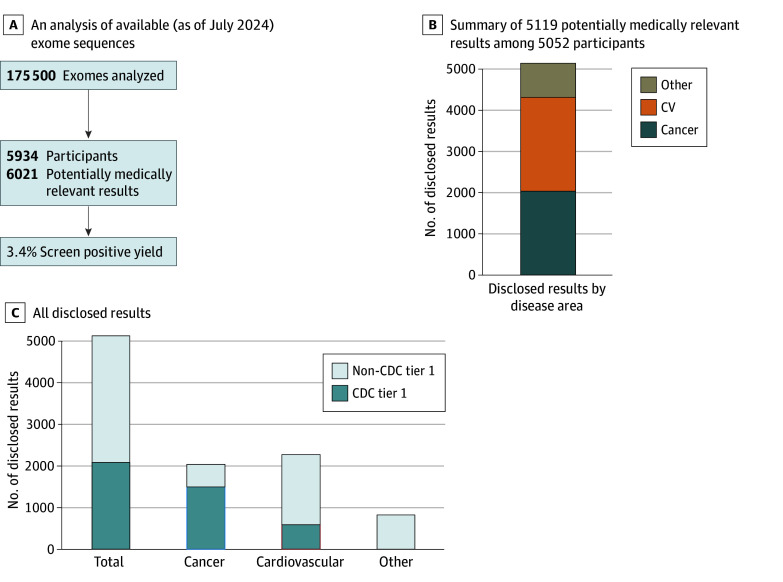
Results From Genomic Screening of Geisinger Program Participants A, Analysis of exome sequences available as of July 2024 (n = 175 500). B and C, Results disclosure to date from all participants with an exome generated (n = 183 822). CDC indicates US Centers for Disease Control and Prevention; CV, cardiovascular.

As of September 2024, 354 957 patients consented to the genomic screening program. Participants had a median (IQR) age of 54 (36-69) years; 194 037 participants (59.7%) had female sex assigned at birth, 9867 participants (3.1%) were Black or African American, 2119 (0.7%) were Asian, and 306 422 (95.7%) were White, and 306 222 (96.2%) had non-Hispanic ethnicity (Table 1). Of program participants, 183 822 had research exome sequencing generated, with 175 500 available for analysis in July 2024, representing approximately 20% of Geisinger’s active patient population. Genomic screening of these participants revealed that 5934 (3.4%) had P/LP variants in potentially medically actionable genes; 87 (0.04% or 1.5% of those with positive results) had results in multiple genes (Figure 1).

**Table 1.** Selected Demographic Characteristics for Cohort

Characteristic	Participants, No. (%)			*P* value^b^
	Total (n = 354 957)	Exome sequenced (n = 183 822)^a^	Results disclosed (n = 5052)	
Ethnicity				
Hispanic or Latino	12 198 (3.8)	4808 (2.8)	118 (2.5)	<.001
Not Hispanic or Latino	306 222 (96.2)	164 028 (97.2)	4678 (97.5)	
Unknown, No.^c^	6525	2776	61	
EHR data unavailable, No.	30 012	12 208	195	
Race				
American Indian or Native Alaskan	851 (0.3)	402 (0.2)	13 (0.3)	<.001
Asian	2119 (0.7)	870 (0.5)	27 (0.6)	
Black or African American	9867 (3.1)	4010 (2.4)	169 (3.5)	
Native Hawaiian or Other Pacific Islander	774 (0.2)	317 (0.2)	8 (0.2)	
Other	268 (0.1)	129 (0.1)	4 (0.1)	
≥2 Races	64 (0.02)	17 (0.01)	0 (0.00)	
White	306 422 (95.7)	164 423 (96.6)	4602 (95.4)	
Unknown, No.^c^	4580	1446	34	
EHR data unavailable, No.	30 012	12 208	195	
Sex assigned at birth				
Female	194 037 (59.7)	104 678 (60.9)	3084 (61.6)	<.001
Male	131 082 (40.3)	67 113 (39.1)	1924 (38.4)	
Unknown, No.^c^	8	5	0	
EHR data unavailable, No.	29 830	12 026	44	
Age^d^				
Median (IQR), y	54 (36-69)	61 (45-73)	59 (44-70)	<.001
Unknown, No.^c^	2156	783	10	
EHR data unavailable, No.	29 830	12 026	44	

Abbreviation: EHR, electronic health record.

^a^ This includes all participants with exome sequences generated. As of July 2024, 175 500 were available for analysis for pathogenic and likely pathogenic variants in genes designated for return though all exomes were screened previously, and pathogenic variants were disclosed to eligible participants.

^b^ *P* values were tabulated by testing the counts of participants in each group by the furthest stage they reached in the genomic screening program.

^c^ Unknown indicates the field was labeled as unknown, whereas EHR data unavailable indicates no access to that specific variable in a patient’s record.

^d^ Age is based on age at last encounter, age at death, or age at program withdrawal.

Of participants with a P/LP variant identified on research exomes available at any point, 5052 were eligible for results disclosure (ie, provided updated consent, had sample available, and were living) and received 5119 clinically confirmed results; 67 participants (1.3%) received results in multiple genes. In participants with results disclosed, the median (IQR) age was 59 (44-70) years and 3084 (61.6%) had female sex assigned at birth (Table 1). Among sequenced participants, there were significant differences in age between those 5052 participants who received results vs those who did not (mean difference: −1.44 years; 95% CI, −1.94 to −0.95 years; *P* <.001); those who received results were, on average, younger. There was no significant difference in sex between these groups (mean difference: −0.67%; 95% CI, −2.05% to 0.71%; *P* = .35). Program participants with genomic findings were significantly different in EHR-documented race but not ethnicity compared with those with exome sequencing who did not receive results. There was a higher rate of Black individuals in the cohort with results disclosed compared with those with exome sequencing who did not receive results, a difference driven by inclusion of *TTR* in the screening list, a gene in which pathogenic variants are observed at increased prevalence in individuals with African ancestry.^20^

**Figure 2.**
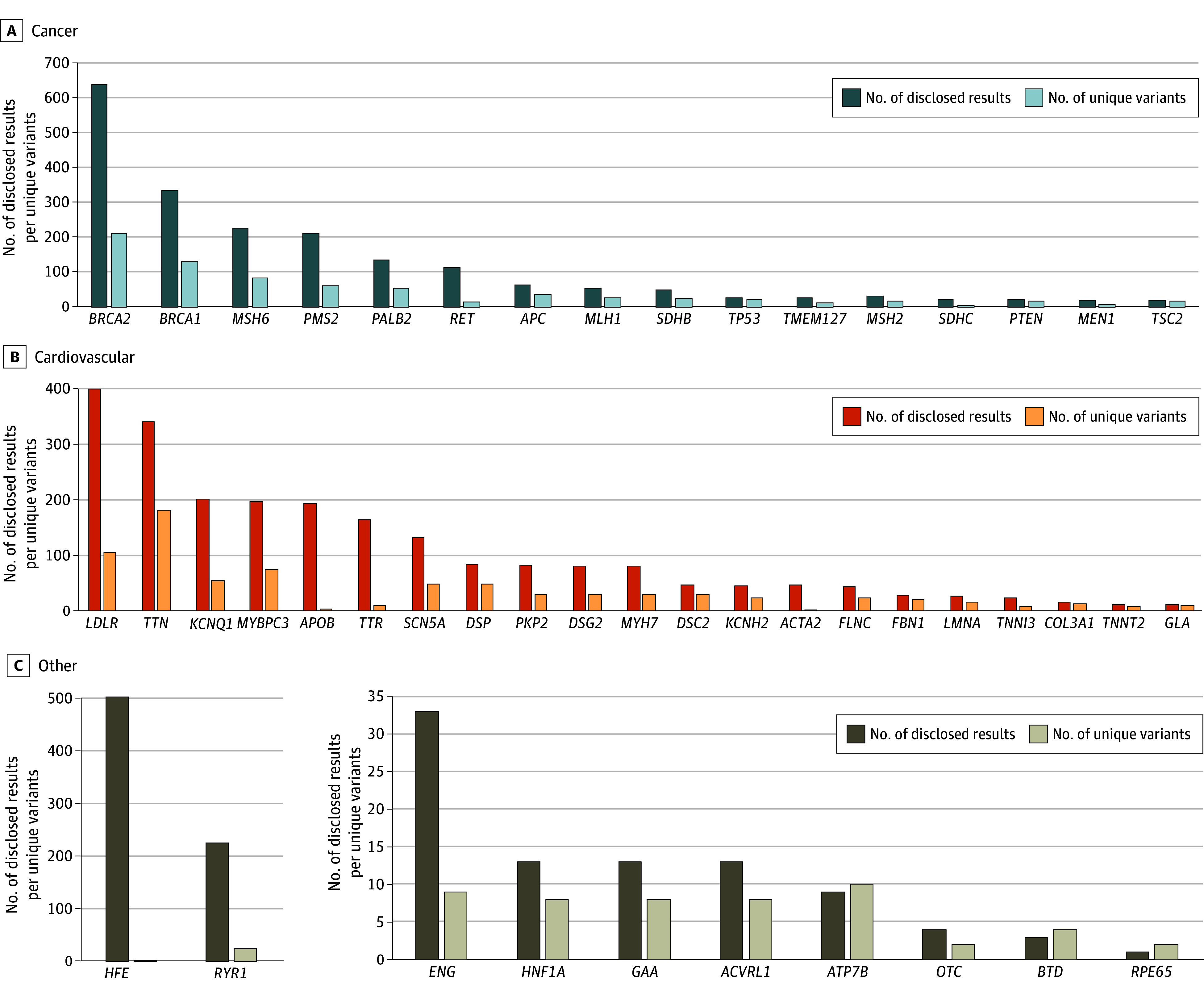
Results Disclosed and Unique Variants by Gene Numbers of disclosed results and unique variants by disease area for genes with at least 10 results disclosed across cancer (A), cardiovascular (B), and other conditions (C).

When examining the types of results disclosed, 2267 results (44.2%) were in cardiovascular genes, 2031 (39.7%) were in cancer genes, and 821 (16.0%) were in other condition genes. Inherited cardiomyopathies accounted for the most cardiovascular results (1040 of 2267 [45.9%]). Results associated with hereditary breast and ovarian cancer (HBOC; 971 of 2031 [47.8%]) or Lynch syndrome (518 of 2031 [25.5%]) accounted for the most results related to cancer. We observed a *BRCA2*-to-*BRCA1* ratio of approximately 2:1 for HBOC, and higher rates of *MSH6* and *PMS2* compared with *MLH1* and *MSH2* for Lynch syndrome (Figure 2). The most common results in the other condition category were hereditary hemochromatosis and malignant hyperthermia, at 9.8% (502 of 5119) and 4.4% (225 of 5119), respectively. Most results (3040 [59.4%]) were in non-CDC Tier 1 genes.

**Figure 3.**
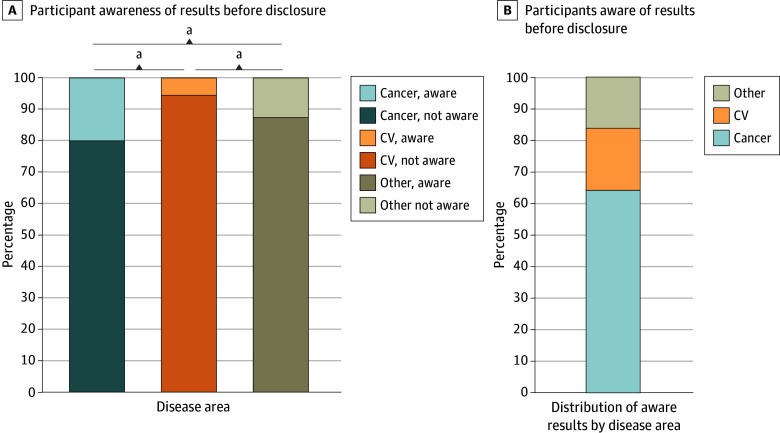
Awareness of Genomic Results at the Time of Disclosure A, Distribution of participants previously unaware (darker color) vs aware (lighter color) of their genomic result at the time of disclosure. B, A total of 627 participants (12.4%) were aware of their risk before sequencing. ^a^*P* < .001.

Of participants who received a result, 4425 (87.6%) were previously unaware of their genomic risk (Figure 3), including 1628 of 2031 (80.1%) with cancer-associated results, 2143 of 2267 (94.5%) with cardiovascular-associated results, and 718 of 821 (87.5%) with other results. These observed differences were statistically significant across all condition groups. A higher proportion of cancer-associated results were known previously compared with cardiovascular-associated results (mean difference, 14.4 percentage points; 95% CI, 12.4-16.4 percentage points; *P* < .001) and other condition results (mean difference, 7.3 percentage points; 95% CI, 4.4-10.2 percentage points; *P* < .001). Other condition results were known previously at higher rates compared with cardiovascular-related results (mean difference, 7.1 percentage points; 95% CI, 4.5-9.6 percentage points; *P* < .001). When restricted to participants with prior knowledge of their results, cancer-associated genes accounted for the highest percentage of results (403 of 630 [64.0%]), followed by cardiovascular-associated results (124 [19.7%]), and hereditary hemochromatosis (66 [10.5%]). Furthermore, those who had a CDC Tier 1 result knew of their genomic risk prior to program participation at higher rates (333 of 2079 [16.0%]) than those who had a non–CDC Tier 1 result (297 of 3040 [9.8%]) (mean difference: 6.2 percentage points; 95% CI, 4.3-8.2 percentage points; *P* < .001).

### Genomic Screening Programs

In surveying the landscape of genomic screening programs, 24 (including the one being studied in this article) met inclusion criteria based on size and data availability (eTable 4 in Supplement 1). Of those, only 6 (25.0%) were disclosing potentially actionable genomic results (Table 2). Programs disclosing these results varied in which results they shared with participants (eg, limiting to CDC Tier 1 results or utilizing an earlier version of the ACMG SF list).

**Table 2.** Large Genomic Programs Reporting Genomic Results to Participants

Program (country)	Current enrollment^a^	Genomic results with potential medical actionability returned^b^
All of Us/NIH (United States)	650 000	ACMG SF version 2.0
Geisinger MyCode Community Health Initiative (United States)	345 073	ACMG SF version 3.2
Estonian Biobank (Estonia)	210 000	ACMG SF 2.0; moderate risk genes (eg, hypolactasia and exfoliative glaucoma, thrombophilia); and cystic fibrosis carrier status
Tohoku University, Tohoku Medical Megabank Organization (Japan)	150 000	Familial hypercholesterolemia
Tapestry with Mayo Clinic (United States)	114 000	CDC Tier 1
Genomics England/100 000 Genomes Project (United Kingdom)	100 000	Diagnostic results related to cancer or rare disease and additional findings (adult and pediatric specific lists)

Abbreviations: ACMG, American College of Medical Genetics and Genomics; CDC, US Centers for Disease Control and Prevention; NIH, National Institutes of Health; SF, Secondary Findings.

^a^ Current enrollment is based on the data available on the International Health Cohorts Consortium website,^19^ the program website, or the literature.

^b^ Results returned includes single-gene genomic results with potential medical relevance and excludes findings such as ancestry, polygenic risk, and pharmacogenomic results.

## Discussion

To our knowledge, our genomic sequencing program was the first large-scale genomic screening program in a health system to disclose potentially medically actionable results to participants who agreed to participate. Having sequenced approximately 20% of Geisinger’s active patient population, identified results in 1 in 30 participants (3.4%), and disclosed more than 5000 results, this program could serve as a model for genomic screening at scale.

The program has demonstrated that genomic screening fills important gaps missed by clinical, indication-based testing and increases identification of at-risk patients. Nearly 90% of participants only learned of their genomic risk due to genomic screening. Other population screening programs have supported these findings.^9,10,21^ The differential rate of prior awareness of genomic findings by result category is likely due to increased awareness of some genes and historical testing practices (eg, long-standing recommendation for cancer genetic testing^22^). Our prior work^5,6,23,24^ demonstrated that a proportion of variant-positive participants had a personal or family history warranting clinical genetics evaluation prior to results disclosure. However, despite robust clinical genetics services at Geisinger, they had not come to clinical attention. For example, 51% of participants with a *BRCA1/2* variant^23^ and 11% with an endocrine tumor syndrome variant^6^ had a history that warranted clinical testing but were not evaluated prior to genomic screening result disclosure. Despite recommendations for evaluation,^25,26,27,28,29^ underutilization of clinical genetic testing has been well described in both hereditary cancer^30^ and cardiovascular disease.^25,26,27,28,29,31,32^ Barriers to clinical evaluation and indication-based genetic testing include limited patient knowledge of their family history, lack of clinician knowledge about genetic conditions and testing recommendations, limited time, little access to genetics services, and concerns about insurance coverage and cost.^33,34^ Although all patients may experience these barriers, they appear particularly acute for patients from underrepresented groups.^35,36,37,38,39^ Innovative strategies, including clinical data review systems, telehealth genetic counseling, chatbots to facilitate personal and family history collection, and pretest educational content, have been suggested to improve identification of at-risk patients.^34,40^ While some of these methods have modestly increased referral rates,^34,40^ they have not addressed all barriers to initial evaluation, referral, and testing.

Since population genomic screening bypasses clinical risk assessment that leverages personal and family history, multiple barriers can be overcome, closing this gap in current clinical, guidelines-based testing practice. Additionally, genomic screening can expand identification of at-risk patients beyond those that meet inadequately sensitive testing criteria. Many participants identified via this screening program did not have a personal or family history consistent with their genomic finding at the time of results disclosure and, as such, did not meet guidelines for clinical testing (eg, 49% with a *BRCA1/2* variant,^23^ 89% with an endocrine tumor syndrome variant^6^). Even with effective family history data collection and efficacious referrals and testing for patients meeting criteria, these patients, some of whom went on to have relevant diagnoses,^6,41^ would remain unidentified without genomic screening due to inadequate testing criteria sensitivity.^6,24,41,42^

For genomic screening to be sustainable and implemented as part of routine clinical care, screening needs to be cost-effective and reimbursed by payers. Modeling studies of screening for *BRCA1/2*, Lynch syndrome, familial hypercholesterolemia, and hereditary hemochromatosis have demonstrated that population genomic screening can be cost-effective.^43,44,45^ Our data indicate that limiting genomic screening to these conditions, as some programs have, however, would only identify a minority of the 1 in 30 participants in this program with a potentially actionable result. The addition of more conditions may improve cost-effectiveness of population genomic screening, if screened genes have sufficient evidence of potential actionability.^43^ Simultaneously, this program and other unselected cohorts have also found that rates of associated phenotypes may be lower in individuals identified via genomic screening.^4,6,8,46,47,48^ As such, not all results previously thought to be highly penetrant and relevant to care may be appropriate for population screening. Data from unselected populations will be needed to further define the penetrance in unselected populations, inform which genes are screened, and shape the counseling and care of those identified. Evidence-based guidelines on what genes and conditions to include in population screening programs are needed.

Prior studies evaluating subsets of the this cohort have examined completion of recommended care in patients identified to have a genomic risk via screening.^5,6,8,24,49^ Across conditions studied, including the CDC Tier 1 conditions,^24,49^ endocrine tumor syndromes,^6^ familial adenomatous polyposis,^8^ and hereditary hemochromatosis,^5^ 38% to 70% of program participants completed recommended risk management following disclosure. Even for participants treated symptomatically prior to result return, genomic screening can impact care. For example, among 96 patients with familial hypercholesterolemia identified via this program, 90% of whom had hyperlipidemia diagnosed before the results disclosure, clinicians intensified medications and patient adherence to medication improved after the genomic result.^50^ Genomic screening also has been shown to have benefit beyond the initially ascertained patient by guiding care for family members identified via cascade testing.^10,11^

Our findings also demonstrate an opportunity to design genomic screening programs that counter the potential for disparities among historically underrepresented groups. Equity in genomic screening programs is challenged by the historical lack of diversity in genomic research studies^51^ and disparities in access to clinical genetic testing that have limited the inclusion of P/LP variant data from underrepresented populations in databases such as ClinVar.^15,35,36,37,38,39^ Although these challenges will continue to impact variant classification, selecting genes in which variants are more prevalent among underrepresented groups, as in the case of the pathogenic *TTR* variant that is common among individuals of African ancestry,^20^ could improve equity in risk identification.

Our review of large-scale programs that generate genomic data highlights opportunities to address these equity concerns and broaden our understanding of genomic screening, clinical utility, and implementation. Even though more than 20 large-scale programs are generating genomic sequences, only 25% disclosed potentially actionable results. Furthermore, incorporating genomic screening into clinical care remains the exception. Enabling genomic screening more broadly would provide substantial opportunities to increase risk identification and enable collection of outcomes data across diverse settings, considerably advancing our understanding of the clinical utility of such screening.

Additional studies that explore potential negative outcomes of population genomic screening will be needed to inform a full assessment of the clinical utility of genomic screening. Data from a subset of Geisinger program participants have begun to explore concerns regarding potential harms of genomic screening, such as negative psychological reactions^52^ and inappropriate health care and associated costs.^53^ Among this subset of participants, result disclosure was associated with low levels of negative emotions and decisional regret after disclosure, with any initial negative psychological impact appearing to wane over time.^54,55^ Furthermore, examination of health care utilization and costs before and after *BRCA1/2* result disclosure via this genomic screening program demonstrated no statistical differences, diminishing concerns about unnecessary care and associated costs.^49^ Yet, we did not collect data on other reported concerns of genomic screening, including potential individual financial costs^56^ or for life or disability insurance discrimination,^33^ which would not be protected against by the Genetic Information Nondiscrimination Act.^57^ Further exploration of psychological harms, health care utilization, costs, and insurability across a larger cohort of patients will be needed to understand the potential for individual- and system-level harms of genomic screening.

### Limitations

This study has several limitations. First, if participation in the genomic screening program was affected by personal or family history, or prior knowledge of a genetic result, this could have affected our findings. Furthermore, we report on our experience from one genomic screening effort in a single health care system that is relatively homogenous in EHR-documented race and ethnicity and did not examine penetrance or changes to participants’ care and outcomes across the more than 5000 participants with a disclosed result. Longitudinal studies from additional systems and more diverse cohorts are needed to fully evaluate the clinical utility and potential actionability of genomic screening across populations. Such studies could determine whether the positive outcomes of genomic screening seen in this and other programs generalize across populations and settings.^4,5,6,7,8,10,11,12,49^

## Conclusions

In this cohort study, 1 in 30 individuals had a potentially actionable genomic finding, but nearly 90% were not aware of this risk. Of large-scale genomic screening programs, only one-quarter disclosed potentially actionable results to participants. While considerations for clinical integration of genomic screening remain,^58^ genomic screening can more effectively identify patients with genomic risk compared with clinical evaluation driven by personal and family history. Continued clinical evidence generation and collaboration among programs are needed to evaluate the risk-benefit balance of genomic screening across diverse settings.

## Notes

Supplement 1. **eTable 1.** Returnable Gene List **eTable 2.** Result Disease Type and CDC Tier 1 Status Compared by Race and Ethnicity Data Availability **eTable 3.** Result Disease Type and CDC Tier 1 Status Compared by Sex Assigned at Birth Data Availability **eTable 4.** Genomic Sequencing Programs With More Than 100,000 Participants, Genomic Sequencing Data, and EHR Data

Supplement 2.
Data Sharing Statement
