# Supplementary material for: Genomic Screening at a Single Health System
Source: JAMA Netw Open. 2025 Mar 17;8(3):e250917. doi: 10.1001/jamanetworkopen.2025.0917 (PMC11915069; doi:10.1001/jamanetworkopen.2025.0917)
Supplement: Supplement 1. — eTable 1. Returnable Gene List eTable 2. Result Disease Type and CDC Tier 1 Status Compared by Race and Ethnicity Data Availability eTable 3. Result Disease Type and CDC Tier 1 Status Compared by Sex Assigned at Birth Data Availability eTable 4. Genomic Sequencing Programs With More Than 100,000 Participants, Genomic Sequencing Data, and EHR Data [file jamanetwopen-e250917-s001.pdf]

Supplemental Online Content

Savatt JM, Kelly MA, Sturm AC, et al. Genomic screening at a single health system. *JAMA Netw Open.* 2025;8(3):e250917. doi:10.1001/jamanetworkopen.2025.0917

eTable 1. Returnable Gene List

eTable 2. Result Disease Type and CDC Tier 1 Status Compared by Race and Ethnicity Data Availability

eTable 3. Result Disease Type and CDC Tier 1 Status Compared by Sex Assigned at Birth Data Availability

eTable 4. Genomic Sequencing Programs With More Than 100,000 Participants, Genomic Sequencing Data, and EHR Data

This supplemental material has been provided by the authors to give readers additional information about their work.

© 2025 Savatt JM et al. *JAMA Network Open*.

eTable 1. Returnable Gene List

Disease	Gene	CDC Tier 1
**Cancer**		
Familial Adenomatous Polyposis	*APC*	-
MUTYH-Associated Polyposis	*MUTYH**	-
Hereditary Breast and Ovarian Cancer Syndrome	*BRCA1*	Y
	BRCA2	Y
	PALB2	-
Hereditary Paraganglioma Pheochromocytoma Syndrome	*MAX*	-
	SDHAF2	-
	SDHB	-
	SDHC	-
	SDHD	-
	TMEM127	-
Juvenile Polyposis	*BMPR1A*	-
	SMAD4[a]	-
Li-Fraumeni Syndrome	*TP53*	-
Lynch Syndrome/HNPCC	*MLH1*	Y
	MSH2	Y
	MSH6	Y
	PMS2	Y
Familial Medullary Thyroid Cancer, Multiple Endocrine Neoplasia, Type 2	*RET*	-
Multiple Endocrine Neoplasia, Type 1	*MEN1*	-
Neurofibromatosis, type 2	*NF2*	-
Peutz-Jeghers Syndrome	*STK11*	-
PTEN Hamartoma Syndrome	*PTEN*	-
Retinoblastoma	*RB1*	-
Tuberous Sclerosis	*TSC1*	-
	TSC2	-
Von Hippel Lindau Syndrome	*VHL*	-
WT1-related Wilms tumor	*WT1*	-
**Cardiovascular**		
Familial Hypercholesterolemia	*APOB*	Y
	LDLR	Y
	PCSK9	Y
Familial Thoracic Aortic Aneurysms and Dissections	*ACTA2*	-
	MYH11	-
	SMAD3	-
	TGFBR1	-
	TGFBR2	-

© 2025 Savatt JM et al. *JAMA Network Open*.

Disease	Gene	CDC Tier 1
**Cardiovascular (cont.)**		
Hereditary Transthyretin-Related Amyloidosis	*TTR*	-
Inherited Arrhythmias	*CALM1*	-
	CALM2	-
	CALM3	-
	*CASQ2**	-
	KCNH2	-
	KCNQ1	-
	RYR2	-
	SCN5A	-
	*TRDN**	-
Inherited Cardiomyopathies	*ACTC1*	-
	BAG3	-
	DES	-
	DSC2	-
	DSG2	-
	DSP	-
	FLNC	-
	GLA	-
	LMNA	-
	MYBPC3	-
	MYH7	-
	MYL2	-
	MYL3	-
	PKP2	-
	PRKAG2	-
	RBM20	-
	TMEM43	-
	TNNC1	-
	TNNI3	-
	TNNT2	-
	TPM1	-
	TTN	-
Marfan Syndrome	*FBN1*	-
Vascular Ehlers-Danlos Syndrome	*COL3A1*	-
**Other conditions**		
Biotinidase Deficiency	*BTD**	-
Hereditary Hemochromatosis	*HFE^*	-
Hereditary Hemorrhagic Telangiectasia	*ACVRL1*	-
	ENG	-

© 2025 Savatt JM et al. *JAMA Network Open*.

Disease	Gene	CDC Tier 1
**Other conditions (cont.)**		
Malignant Hyperthermia Susceptibility	*CACNA1S*	-
	RYR1	-
Maturity-Onset Diabetes in the Young	*HNF1A*	-
Ornithine Transcarbamylase Deficiency	*OTC*	-
Pompe Disease	*GAA**	-
Retinopathy	*RPE65**	-
Wilson Disease	*ATP7B**	-

*Autosomal recessive disease, only comp heterozygous/homozygous variants to be reported; ^Genotyping for p.Cys282Tyr homozygotes only; [a]*SMAD4* is also associated with Hereditary Hemorrhagic Telangiectasia but was categorized as a cancer gene given its association with juvenile polyposis.

© 2025 Savatt JM et al. *JAMA Network Open*.

eTable 2. Result Disease Type and CDC Tier 1 Status Compared by Race and Ethnicity Data Availability

		Missing Race/Ethnicity		Not Missing		Comparison
		(n=269)		(n=4,850)		
**Disease Area**	Cancer	89	45.64%	1,942	39.44%	p=0.12
	Cardio	83	42.56%	2,184	44.35%	
	Other	23	11.79%	798	16.21%	
**CDC Tier 1 Status**	Yes	100	51.28%	1,979	40.19%	p=0.003
	No	95	48.72%	2,945	59.81%	

Note: This table sums to the total number of results (n=5,119) instead of the number of patients. This is because Disease Area and CDC Tier 1 Status are by result not by patient (e.g., a patient with a *BRCA1* and *TTN* result will be in both the cancer and cardio categories).

© 2025 Savatt JM et al. *JAMA Network Open*.

eTable 3. Result Disease Type and CDC Tier 1 Status Compared by Sex Assigned at Birth Data Availability

		Missing Sex (n=44)		Not Missing (n=5,075)		Comparison
**Disease Area**	Cancer	19	43.18%	2,012	39.65%	p=0.245
	Cardio	22	50.00%	2,245	44.24%	
	Other	3	6.82%	818	16.12%	
**CDC Tier 1 Status**	Yes	20	45.45%	2,059	40.57%	p=0.615
	No	24	54.55%	3,016	59.43%	

Note: This table sums to the total number of results (n=5,119) instead of the number of patients. This is because Disease Area and CDC Tier 1 Status are by result not by patient (e.g., a patient with a *BRCA1* and *TTN* result will be in both the cancer and cardio categories).

© 2025 Savatt JM et al. *JAMA Network Open*.

eTable 4. Genomic Sequencing Programs With More Than 100,000 Participants, Genomic Sequencing Data, and EHR Data

Program (Country)	Current Enrollment	Returning Potentially Medically Actionable Genomic Results[a]
Danish National Biobank (Denmark)	4,000,000	No
Dementias Platform UK (UK)	3,600,000	No
Million Veteran Program (USA)[a]	1,000,000	No
All of Us / NIH (USA)	650,000	**Yes**
National Biobank of Korea (South Korea)	650,000	No
China Kadoorie Biobank (China)	512,000	No
UK Biobank (UK)	501,479	No
Kaiser Permanente Research Bank (KPRB) (USA)	419,000	No
Geisinger MyCode Community Health Initiative (USA)	345,073	**Yes**
Canadian Partnership for Tomorrow's Health (Canada)	345,000	No
45 and Up Study (Australia)	267,000	No
Penn Medicine Biobank (USA)	264,194	No
BioVU Vanderbilt (USA)	244,000	No
HUNT 70+, The HUNT Study (Norway)	240,000	No
Multiethnic Cohort Study (USA)	215,000	No
CONSTANCES (France)	215,000	No
Estonian Biobank (Estonia)	210,000	**Yes**
South Asia Biobank (India, Sri Lanka, Bangladesh)	180,000	No
Tohoku University, Tohoku Medical Megabank Organization (Japan)	150,000	**Yes**
Shanghai Men and Women's Health Studies (China)	136,000	No
Northern Sweden Health and Disease (Sweden)	125,000	No
Tapestry with Mayo Clinic (USA)	114,000	**Yes**
UK Blood Donors Cohorts (UK)	100,000	No
Genomics England/ 100,000 Genomes Project (UK)	100,000	**Yes**

[a] Several programs have launched sub-studies to enable return of results. These are not captured as returning results across the program. Green denotes programs returning results with potential medical actionability.

© 2025 Savatt JM et al. *JAMA Network Open*.
