# Supplementary material for: Genomic Screening at a Single Health System
Source: JAMA Netw Open. 2025 Mar 17;8(3):e250917. doi: 10.1001/jamanetworkopen.2025.0917 (PMC11915069; doi:10.1001/jamanetworkopen.2025.0917)
Supplement: Supplement 2. — Data Sharing Statement [file jamanetwopen-e250917-s002.pdf]

# Data Sharing Statement

Savatt. Genomic Screening at a Single Health System. *JAMA Netw Open*. Published March 17, 2025. doi:10.1001/jamanetworkopen.2025.0917

**Data**
**Data available:** Yes
**Data types:** Deidentified participant data
**How to access data:** Variant level data for the likely pathogenic/pathogenic variants reported in this paper can be requested by sending an email to MyCodeResults@geisinger.edu.
**When available:** With publication

**Supporting Documents**
**Document types:** None

**Additional Information**
**Who can access the data:** Data will be available to qualified academic noncommercial researchers
**Types of analyses:** for a specified purpose
**Mechanisms of data availability:** after approval of a proposal with a signed data access agreement
